# PHF6 loss reduces leukemia stem cell activity in an acute myeloid leukemia mouse model

**DOI:** 10.1186/s12935-024-03265-w

**Published:** 2024-02-09

**Authors:** Shengnan Yuan, Mingming Gao, Yizhou Wang, Yanjie Lan, Mengrou Li, Yuwei Du, Yue Li, Wen Ju, Yujin Huang, Ke Yuan, Lingyu Zeng

**Affiliations:** 1 School of Medical Technology, Xuzhou Medical University, Xuzhou, Jiangsu China; 2 Blood Diseases Institute, Xuzhou Medical University, No. 209, Tongshan Road, Xuzhou, Jiangsu 221004 China; 3 Key Laboratory of Bone Marrow Stem Cell, Xuzhou, Jiangsu China; 4 Department of Hematology, The Affiliated Hospital of Xuzhou Medical University, Xuzhou, Jiangsu China; 5 Department of Neuro-oncology, Cancer Center, Beijing Tiantan Hospital, Capital Medical University, Beijing, 100071 China

**Keywords:** *PHF6*, Acute myeloid leukemia (AML), Cell differentiation, Leukemia stem cells (LSCs)

## Abstract

Acute myeloid leukemia (AML) is a malignant hematologic disease caused by gene mutations and genomic rearrangements in hematologic progenitors. The PHF6 (PHD finger protein 6) gene is highly conserved and located on the X chromosome in humans and mice. We found that *PHF6* was highly expressed in AML cells with *MLL* rearrangement and was related to the shortened survival time of AML patients. In our study, we knocked out the *Phf6* gene at different disease stages in the AML mice model. Moreover, we knocked down PHF6 by shRNA in two AML cell lines and examined the cell growth, apoptosis, and cell cycle. We found that PHF6 deletion significantly inhibited the proliferation of leukemic cells and prolonged the survival time of AML mice. Interestingly, the deletion of PHF6 at a later stage of the disease displayed a better anti-leukemia effect. The expressions of genes related to cell differentiation were increased, while genes that inhibit cell differentiation were decreased with PHF6 knockout. It is very important to analyze the maintenance role of PHF6 in AML, which is different from its tumor-suppressing function in T-cell acute lymphoblastic leukemia (T-ALL). Our study showed that inhibiting PHF6 expression may be a potential therapeutic strategy targeting AML patients.

**Supplementary Information:**

The online version contains supplementary material available at 10.1186/s12935-024-03265-w.

## Introduction

Acute myeloid leukemia (AML) is one of the most common malignant diseases of hematopoietic stem (progenitor) cells, characterized by the accumulation of immature myeloid progenitors in the bone marrow (BM) and peripheral blood (PB) of patients [1]. Most of the patients have severe symptoms, such as hematopoietic failure, anemia, infection, and organ infiltration [2]. The overall survival rate of adult AML patients with *MLL* gene rearrangement is less than 20%. Elderly patients over 60 years old cannot tolerate intensive chemotherapy, so the treatment options are limited, and the survival rate is less than 15% [3]. Existing research shows that AML is a group of polygenic diseases resulting from multi-step gene mutation accumulation. AML is driven by an oncogenic driver gene and modulated jointly by secondary gene mutations during disease development [4]. 11q23/*MLL* rearrangement is an important driver gene of AML [5]. It has been proven that *MLL*::*AF9* gene rearrangement can effectively promote the transformation of normal hematopoietic progenitor cells into leukemia cells [6, 7]. The frequency of *MLL* rearrangement is about 10% in adult AML and is as high as 16 − 21% among children with AML [8–10]. *MLL* rearrangement is closely related to drug resistance and relapse of leukemia and is an important factor leading to poor prognosis of AML [11].

The MLL partner gene mediates different gene expressions, phenotypes, and clinical outcomes. Therefore, the MLL partner gene is the primary cause of heterogeneity among MLL-rearranged AML. Interestingly, even with AML induced by the same *MLL* rearrangement, patients’ clinical manifestations, drug reactions, and disease severity are inconsistent [12]. In the process of AML, the subsequent acquired gene abnormalities lead to heterogeneity, directly affecting the development of AML. Gene mutations such as NPM1, FLT3, DNMT3A, IDH1/2, TET2, PHF6, TP53, WT1, and NRAS acquired at different stages of the disease often impact the prognosis of patients [12, 13]. The PHF6 (PHD finger protein 6) gene is located on the X chromosome (Xq26–27), which is highly conserved in vertebrates, both in humans and mice [14]. PHF6 belongs to the PHD superfamily. The common feature of this family is that it contains PHD zinc finger structures [15]. The mutation rate of PHF6 in acute T-lymphocyte leukemia (T-ALL) is about 20–30% [16, 17]. In AML, the mutation frequency of PHF6 is about 3% [18]. PHF6 mutations are mainly in the form of inactivation, including frameshift, truncation, and nonsense, concentrated primarily on the second PHD domain [19]. Most of these mutations are found in male patients [17]. Therefore, we constructed a PHF6 knockout model using male mice.

It was found that hematopoietic stem cells (HSCs) with PHF6 deficiency have higher proliferation and reconstruction capacity than the wild-type HSCs [20]. PHF6 deficiency alone is insufficient to induce abnormal hematopoietic transformation [16]. Research has shown that PHF6 deletion can significantly accelerate T-ALL progression caused by NOTCH1 and JAK3^M511I^ [20, 21]. Moreover, Pawar A et al. found that the loss of PHF6 contributed to the leukemogenesis of Hoxa9-driven AML [22]. While Hou et al. demonstrated that PHF6 loss inhibited the initiation of RUNX1-ETO 9a-driven myeloid leukemia [23]. In a BCR-ABL1-driven B-ALL murine model, Meacham et al. also showed that the knockdown of PHF6 impaired the B-ALL cell growth [24]. These findings have suggested that PHF6 may act as an oncogene or a tumor suppressor depending on the specific context induced by different driver mutations. In clinical cases, leukemia often occurs by a driver gene mutation, and other mutations are acquired at different stages during disease progression [4, 13]. These subsequent mutations result in the heterogeneity of AML and have an important impact on the development and outcome of the disease [1]. PHF6 plays an important role in the occurrence and development of leukemia [20, 25]. Therefore, we constructed an *MLL::AF9*-induced AML mouse model to study how PHF6 deficiency affects the AML progression. Here, we used *Mx1-Cre* to knock out the *Phf6* gene in the hematopoietic system time-controllably.

In the study, we aimed to identify the function of PHF6 in the context of *MLL::AF9*-induced AML in vivo using mouse models. We found PHF6 deficiency can induce myeloid cell differentiation and thus suppress AML progression.

## Materials and methods

### Construction of leukemia mouse models

The mice were kindly provided by Professor Weiping Yuan [21]. Exon 4 to exon 5 of *Phf6* were flanked by two LoxP sequences using the homologous recombination technique. The mice were mated with *Mx1-Cre* transgenic mice to generate the offspring with LoxP and *Mx1-Cre*. Lineage-negative (Lin^−^) cells from BM of male mice were enriched (Miltenyi Biotec, Bergisch Gladbach, Germany) and cultured in complete medium (Dulbecco’s modified Eagle medium) with 15% fetal bovine serum (FBS), 50 ng/ml SCF, 10 ng/ml IL-3 and 10 ng/ml IL-6 (PeproTech, New Jersey, USA). The *MLL::AF9*-IRES-GFP retroviral vector was transfected into 293T cells, and the virus was harvested after 48/72 hours. Lin^−^ cells were transfected with the *MLL::AF9*-GFP virus. 1 × 10^6^ GFP^+^ cells were injected i.v. (intravenous injection) into male mice to establish the *Mx1-Cre; Phf6*^*fl/y*^ AML mouse model.

### PHF6 knockout induced by pIpC

Polyriboinosinic acid/polyribocytidylic acid (pIpC; InvivoGen) was dissolved in PBS at 1 mg/ml. To activate the *Mx1-Cre* transgene, pIpC was injected with 10 mg/kg of mice thrice every other day through subcutaneous injection. Mice were housed in a specific pathogen-free (SPF) animal facility at Xuzhou Medical University.

### Construction of PHF6 knockdown cell line model

The shRNA targeting PHF6 was designed using online software (http://dharmacon.gelifesciences.com). DNA containing the shRNA sequence or the scramble control sequence was subcloned into the SF-LV-GFP vector with XhoI and EcoRI, and single clone was picked and verified by Sanger sequencing. The shRNA sequence is GCTGAGTTTGACATGTTGATA. The scramble control sequence is TCCGCAGGTATGCACGCGTG. For lentivirus production, 293T cells were transfected with shRNA containing SF-LV-GFP plasmids and the helper plasmid pMD.G and psPAX2 with Lipofectamine 2000 (11,668,019, Invitrogen, USA). Virus-containing supernatant medium was collected 2 days after transfection. AML cells were then transduced with scramble or shRNA lentivirus supernatant. After 2 days of culture, the transduced cells were collected, and the GFP^+^ cells were sorted using the flow cell sorter. We confirmed the knockdown status of PHF6 at both the mRNA and protein levels using QPCR and WB, respectively.

### Flow cytometry analysis

Single-cell suspensions were prepared from PB, BM, and spleen. Cells were stained with the flow antibodies for 30 min in ice-cold PBS supplemented with 2% FBS. The acquisition was performed using an LSR Fortessa™ cell analyzer (BD Biosciences, New Jersey, USA) and analyzed with FlowJo, version 10.1 (Tree Star). The flow antibodies used are listed in the supplementary material.

### Statistical analysis

Data were processed in Microsoft Excel (Microsoft, Redmond, WA, USA) or GraphPad Prism (GraphPad Software, La Jolla, CA, USA) software. We assumed normality and equal variance distribution between groups and analyzed the data with Student’s *t*-test. *P* < 0.05 was considered statistically significant (*p < 0.05, **p < 0.01, ***p < 0.001, ****p < 0.0001). Kaplan-Meier curves were used to plot survival, and significance differences were calculated with the log-rank test.

## Results

### *PHF6* is highly expressed in AML patients with *MLL* rearrangement

**Fig. 1:**
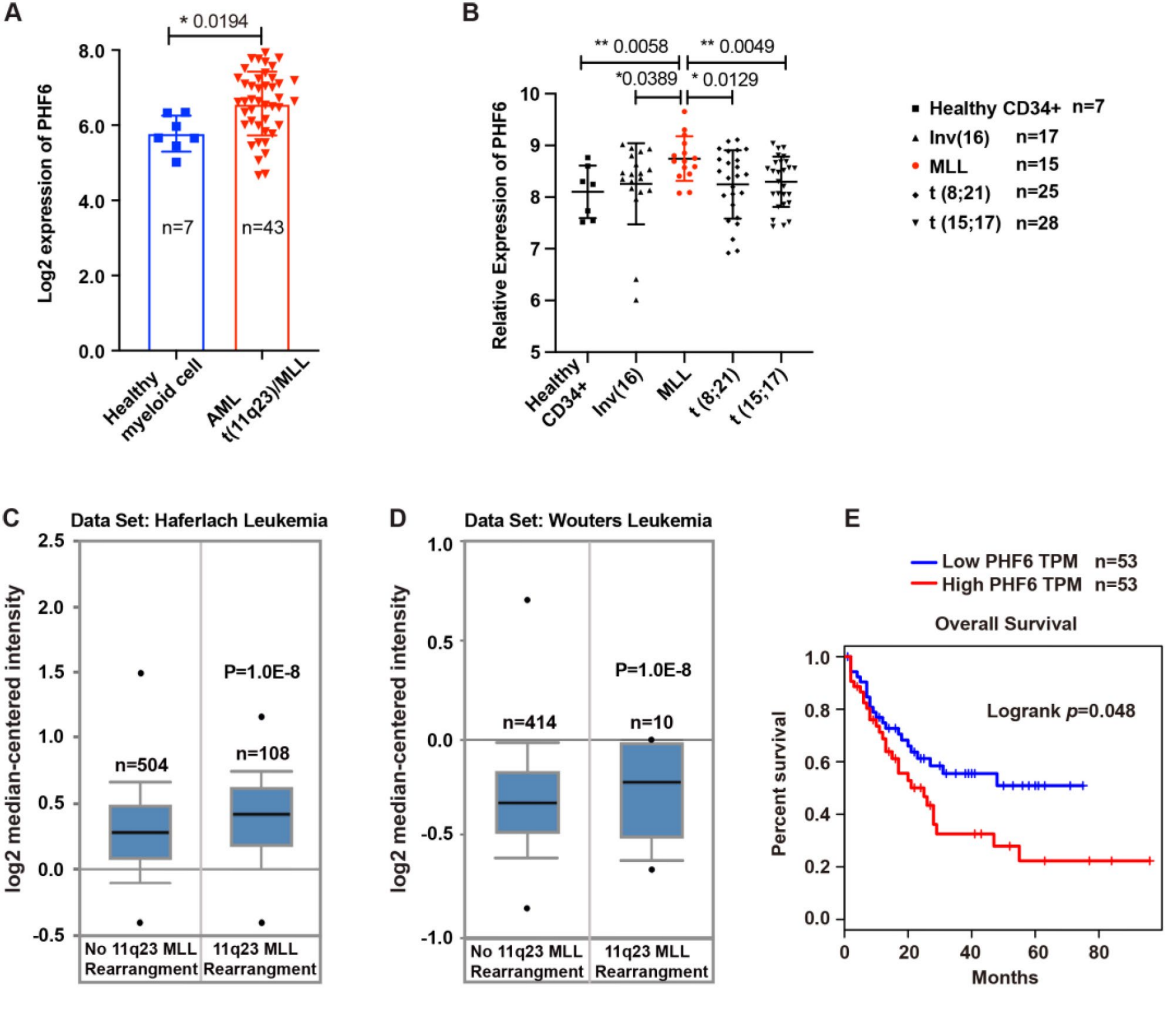
High expression of PHF6 in AML patients with *MLL* rearrangement. (**A**) Comparison of PHF6 expression in AML patients with *MLL* rearrangement (11q23/MLL) and in healthy myeloid cells (Data from bloodspot dataset, www.bloodspot.eu); (**B**) Comparison of PHF6 expression in AML cases with MLL rearrangements (11q23/MLL) and without MLL rearrangements (non-MLL). Inv [16], t (8;21) and t (15;17) are AML subtypes (Published microarray dataset [26]); (**C**-**D**) PHF6 was highly expressed in AML patients with *MLL* rearrangements (Data from oncomine dataset, https://www.oncomine.org); (**E**) Survival analysis of AML patients (Data from gepia dataset, http://gepia.cancer-pku.cn/detail.php?gene=PHF6). 106 AML patients were ranked from high to low based on PHF6 expression, and the median was determined. The 53 AML patients above the median of PHF6 expression were classified as high expression group; The 53 AML patients below the median of PHF6 expression were classified as low expression group. Log-rank (Mantel–Cox) test was used. A two-sided *P* < 0.05 was considered statistically significant

To study the role of PHF6 in AML, we analyzed the expression of PHF6 in normal hematopoietic cells and AML cells with or without *MLL* rearrangement. We found PHF6 was higher in AML patients with *MLL* rearrangement compared to healthy cells (Fig. 1A and Supplementary Fig. 1A-B). Moreover, PHF6 expression in AML patients with *MLL* rearrangement was higher than that in patients without *MLL* rearrangement in different AML databases (Fig. 1B-D). We further examined the expression of PHF6 by western blot in AML cell lines with or without MLL rearrangement. NOMO-1, MV4-11, MOLM13 and THP-1 harbored the MLL rearrangements, and had relatively higher PHF6 expression compared to other non-MLL rearrangements cells (K562, HL60, KASUMI-1 and OCI-AML3) (Supplementary Fig. 1C-D). By analyzing the relationship between the expression of PHF6 and the prognosis of AML patients, we found that the survival time of AML patients with high PHF6 expression was shortened (Fig. 1E). These results suggested that the increased expression of PHF6 was consistent with AML aggressiveness.

### Low expression of *PHF6* inhibits the proliferation of AML cells

**Fig. 2:**
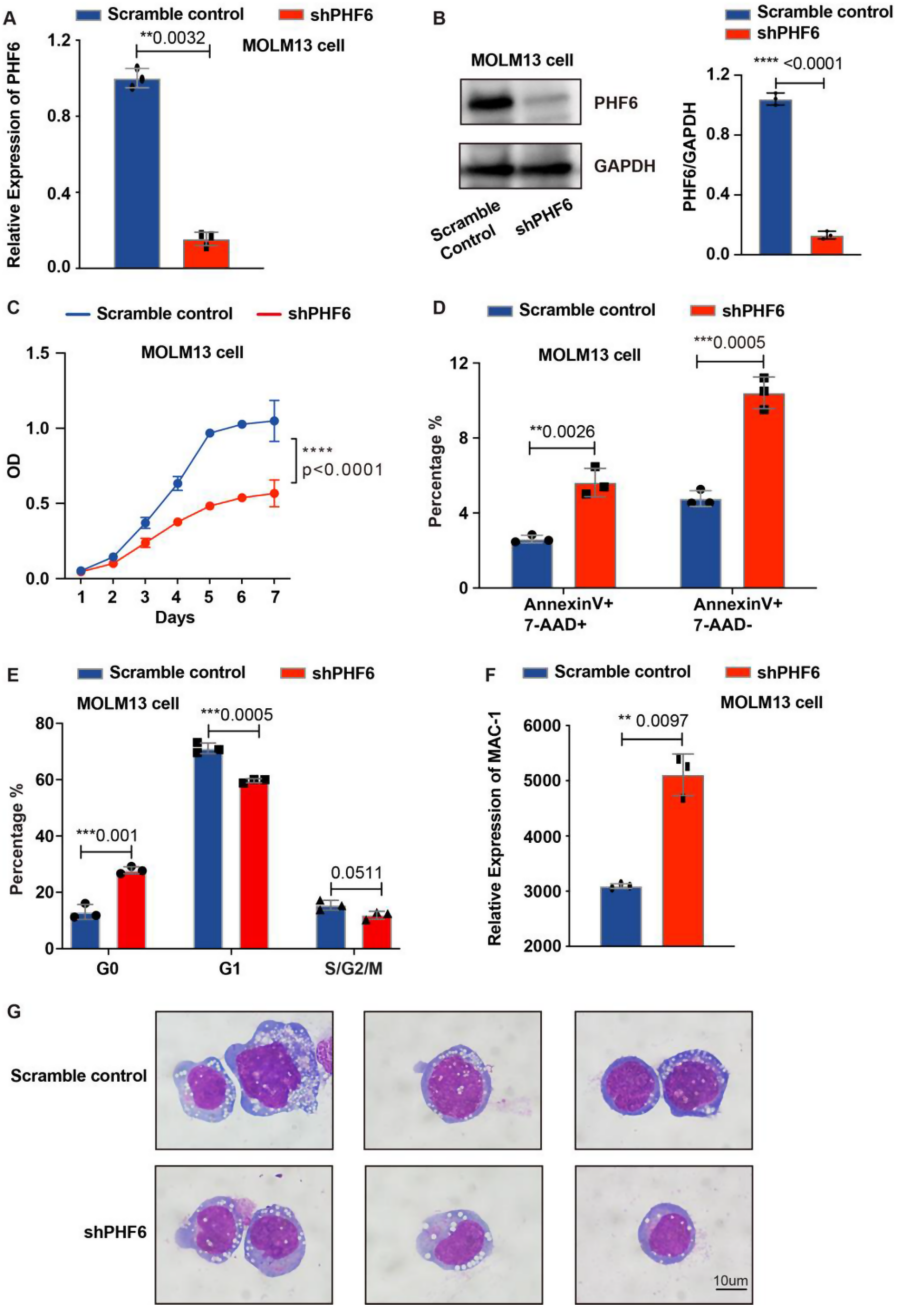
Low expression of PHF6 inhibits the proliferation of MOLM13 cell. (**A**) PHF6 expression in MOLM13 cells analyzed by QPCR. *n* = 3; (**B**) PHF6 expression in MOLM13 cells determined by Western Blot (Left) and analyzed by densitometry plots (Right); (**C**) Examination of cell proliferation by CCK-8. *n* = 6, Student’s *t*-test; (**D**) Examination of cell apoptosis. *n* = 3, Student’s *t*-test; (**E**) Examination of cell cycle. *n* = 3, Student’s *t*-test; (**F**) The expression of MAC-1 examined by flow cytometry in MOLM13 cell. *n* = 3; (**G**) Wright-Giemsa staining of PHF6 knockdown and the control cells. *p < 0.05, **p < 0.01, ***p < 0.001, ****p < 0.0001

To further verify the role of PHF6 in AML, we used shRNA to knock down the *PHF6* gene in two human AML cell lines containing *MLL::AF9* rearrangement (MOLM13 and THP1). We confirmed the knockdown status of PHF6 in MOLM13 cell by qPCR and WB (Fig. 2A-B). The cell proliferation of *PHF6* knockdown group was decreased compared to the MOLM13 control group (Fig. 2C). The percentage of apoptotic cells was increased when *PHF6* was knocked down (Fig. 2D). By analyzing the cell cycle in the two groups, we found that the percentage of G0 cells was significantly increased and dividing cells (G1, S/G2/M) were decreased in the *PHF6* knockdown group (Fig. 2E and Supplementary Fig. 2A). We examined the MAC-1 expression by flow to assess the differentiation of AML cells. We found that the expression of MAC-1 was higher in MOLM13 cell with PHF6 knockdown compared to the scramble control (Fig. 2F). Wright-Giemsa staining was performed to discern the morphological characteristics of AML cells. MOLM13 cells with PHF6 knockdown showed smaller size, slight nuclear segmentation and reduced nucleo-cytoplasmic ratio when compared with the scramble control (Fig. 2G). We confirmed the knockdown of PHF6 in THP1 cells by qPCR and WB (Supplementary Fig. 2B-C). We also examined the phenotype in THP1 cells, and we got the same results of decreased cell proliferation, increased apoptosis, and inactive cell cycle when *PHF6* was knocked down (Supplementary Fig. 2D-F). MAC-1 expression was higher in THP1 cell with PHF6 knockdown (Supplementary Fig. 2G). Smaller size, slight nuclear segmentation, and reduced nucleo-cytoplasmic ratio were seen in THP1 cell with PHF6 knockdown when compared with the scramble control (Supplementary Fig. 2H).

### *Phf6* deletion suppresses *MLL::AF9*-induced AML progression

**Fig. 3:**
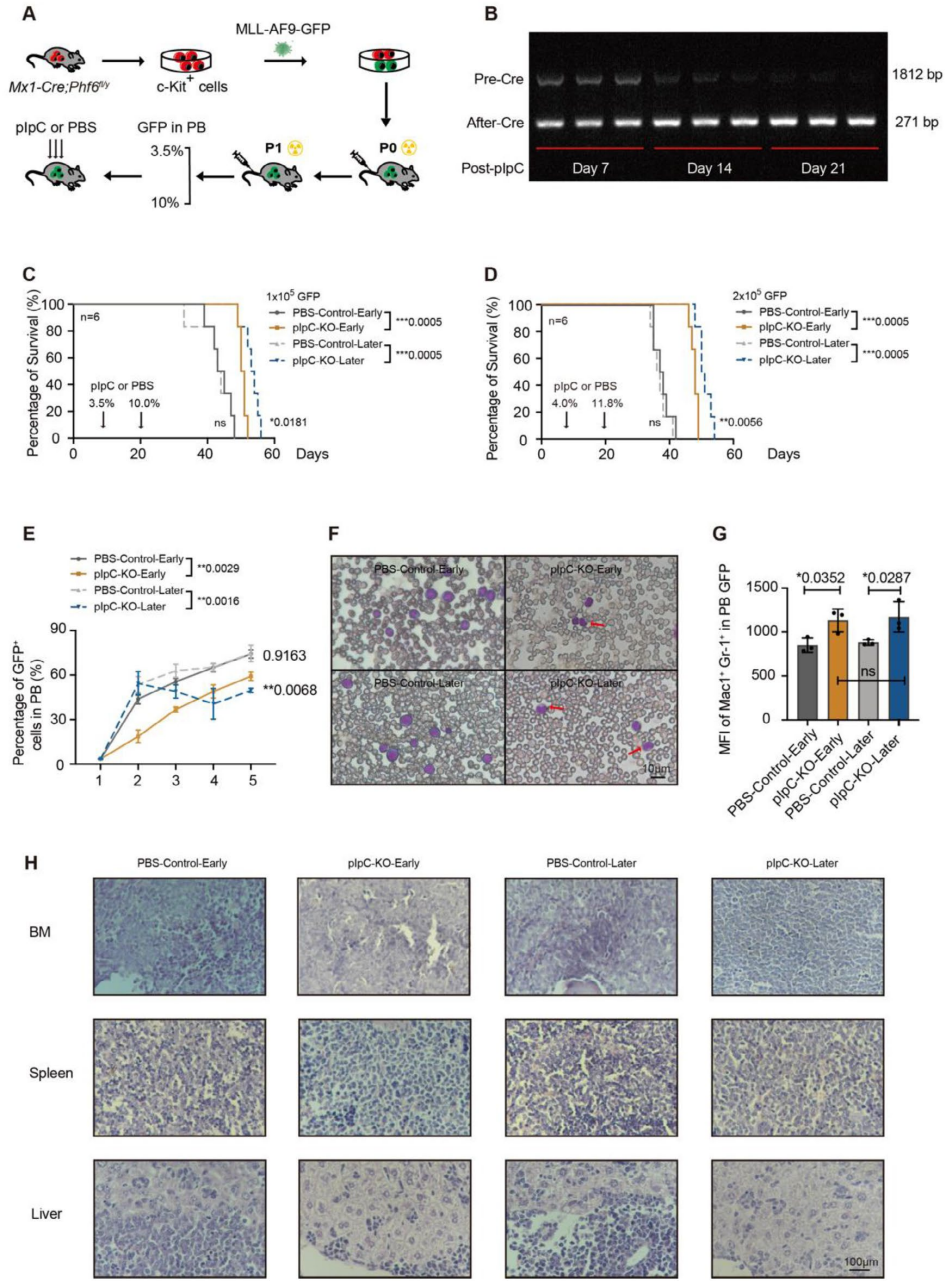
*Phf6* deletion suppresses *MLL::AF9*-induced AML progression. (**A**) Experimental scheme of establishing *Phf6* KO AML model; (**B**) *Phf6* deletion was confirmed by molecular gel electrophoresis of DNA. *n* = 3; (**C**-**D**) Survival analysis of mice transplanted with 1 × 10^5^ (**C**) and 2 × 10^5^ GFP^+^ cells (**D**). *n* = 6, significance differences were calculated with the log-rank test; (**E**) The frequency of GFP^+^ cells in PB was examined at different times post pIpC. The first arrow represents when the PB GFP^+^ cells reached 3.5% and pIpC injection. The second arrow represents when the PB GFP^+^ cells reached 10% and pIpC injection. *n* = 3, Student’s *t*-test; (**F**) Wright–Giemsa staining of PB cells; (**G**) The expression level of Mac-1^+^ Gr-1^+^ in GFP^+^ cells. *n* = 3, Student’s *t*-test; (**H**) Hematoxylin-eosin (HE) staining of BM, spleen, and liver. *p < 0.05, **p < 0.01, ***p < 0.001, ****p < 0.0001

To evaluate the role of *Phf6* in AML, we used a retrovirus-mediated transduction system to build an *MLL::AF9*-induced AML model. We sorted c-Kit^+^ cells from BM of *Mx1-Cre; Phf6*^*fl/y*^ male mice and transfected cells with the *MLL::AF9* virus. Leukemia cells isolated from mouse models of P0 generation were transplanted into male recipients (Fig. 3A). The pIpC or PBS was injected subcutaneously when GFP^+^ cells in peripheral blood (PB) reached 3.5% or 10% respectively (Supplementary Fig. 3A-B). *Phf6* deletion by pIpC injection when the PB GFP^+^ cells attained 3.5% was named pIpC-KO-Early, and its control group was named PBS-Control-Early. *Phf6* deletion by pIpC injection when the PB GFP^+^ cells attained 10% was named pIpC-KO-Later, and its control group was named PBS-Control-Later. *Phf6* deletion was confirmed by molecular gel electrophoresis of DNA extracted from mouse PB cells on Days 7, 14, and 21 after being treated with pIpC (Fig. 3B). The missing long band (1812 bp) and the emerging short band (271 bp) indicated the successful deletion of *Phf6* gene (Fig. 3B). Further, we examined PHF6 protein in BM leukemia cells at Day 21 post pIpC to confirm the deletion of PHF6 through the method of western blot (Supplementary Fig. 3C). The survival time of two KO groups was longer than that in the control groups. Interestingly, the survival time of the pIpC-KO-Later group was longer than that of the pIpC-KO-Early group (Fig. 3C-D). To ensure the accuracy of the survival time experiment of leukemic mice, we sorted two doses of leukemia cells (1 × 10^5^ or 2 × 10^5^ GFP^+^ cells) for transplantation in our study, and we got the same results of survival time from transplantation of the two doses (Fig. 3C-D). We examined the percentage of GFP^+^ leukemia cells after pIpC injection. We found that the percentages of PB GFP^+^ cells in the two *Phf6* KO groups were lower than their corresponding control groups, and the percentage of GFP^+^ cells in the pIpC-KO-Later mice was lower than that in the pIpC-KO-Early group (Fig. 3E). The Wright–Giemsa staining of PB cells showed the morphological characteristics of leukemia cells. We found that leukemia cells in the two *Phf6* KO groups showed a tendency towards maturity with smaller and lobulated nuclei compared to the controls with larger cell size and round nuclear contours (Fig. 3F). We further examined the expression level of Mac-1 and Gr-1 in PB GFP^+^ cells. We found that the expression of Mac-1^+^/Gr-1^+^ was increased in the two *Phf6* KO groups compared with their PBS control counterparts, which suggested an increase in cell maturity of the *Phf6* KO groups (Fig. 3G). The infiltration degree of leukemia cells in the bone, spleen, and liver were reduced in the two *Phf6* KO groups compared to the control mice (Fig. 3H). These results suggested a potential role of *Phf6* in AML maintenance.

### *Phf6* deletion suppresses AML in the serial transplantations

**Fig. 4:**
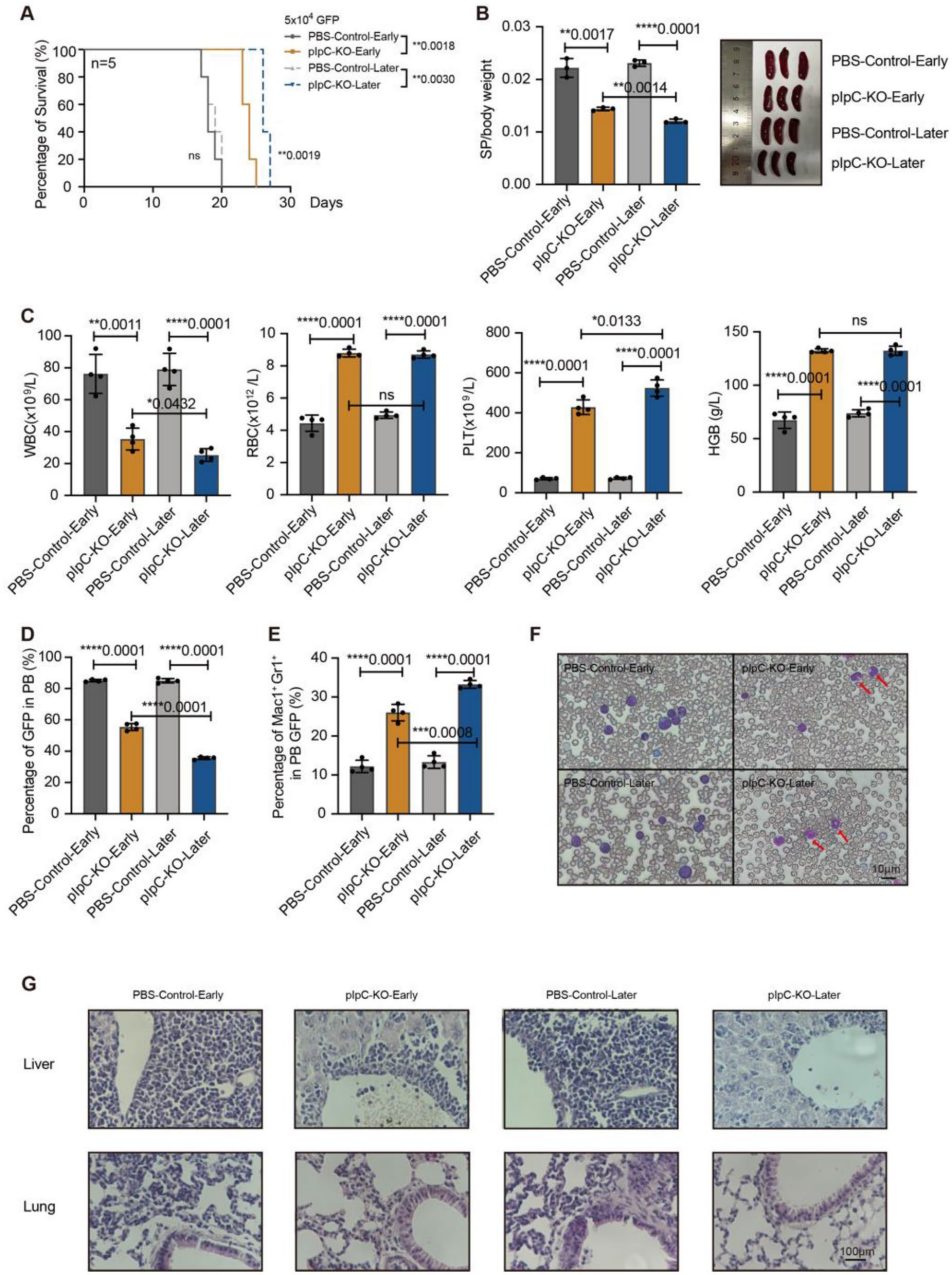
*Phf6* deletion suppresses AML in the serial transplantation. (**A**) Survival time analysis of P2 generation. *n* = 5, significance differences were calculated with the log-rank test; (**B**) Spleen/body weight index analysis. *n* = 3, Student’s *t*-test; (**C**) The blood routine test of WBCs, RBCs, PLTs, and HGB. *n* = 4, Student’s *t*-test; (**D**) Analysis of GFP^+^ cells in the PB. *n* = 4, Student’s *t*-test; (**E**) Analysis of Mac-1^+^/Gr-1^+^ cells in the PB. *n* = 4, Student’s *t*-test; (**F**) Wright–Giemsa staining of PB cells; (**G**) HE staining of liver and lung from AML recipients. *p < 0.05, **p < 0.01, ***p < 0.001, ****p < 0.0001

We sorted GFP^+^ cells from AML mice of P1 generation and transplanted 5 × 10^4^ GFP^+^ cells to recipient mice. All mice succumbed to AML within 30 days. We analyzed the mice phenotype on day 18 after transplantation. The mice transplanted with *Phf6* KO cells had significantly longer survival time than the corresponding controls (Fig. 4A). The survival time of pIpC-KO-Later group was longer than that in the pIpC-KO-Early group (Fig. 4A). The spleen/body weight index of *Phf6* KO mice was much lower than that of PBS control mice and the spleen index in pIpC-KO-Later group was the smallest (Fig. 4B). The blood routine test showed less white blood cells (WBCs), more red blood cells (RBCs), platelets (PLTs), and hemoglobin (HGB) in *Phf6* KO mice (Fig. 4C). The percentage of GFP^+^ cells was lower in PB of *Phf6* KO mice than the PBS controls, and the pIpC-KO-Later group had less GFP^+^ cells than the pIpC-KO-Early group (Fig. 4D and Supplementary Fig. 4A). AML cells expressed with double positive markers of Mac-1 and Gr-1 of *Phf6* KO mice were higher than the counterparts respectively, and Mac-1^+^/Gr-1^+^ cells in the pIpC-KO-Later group were higher than the pIpC-KO-Early group (Fig. 4E and Supplementary Fig. 4B). Wright–Giemsa staining showed myeloid differentiation of AML cells in *Phf6* KO mice when compared to the controls (Fig. 4F). The infiltration of leukemia cell in the liver and lung were decreased in the two *Phf6* KO groups than the control mice (Fig. 4G). The results from the serial transplantation suggested that the *Phf6* KO mice showed less aggressive leukemia phenotypes.

### *Phf6* deficiency decreases the activity of leukemia stem cells in AML

**Fig. 5:**
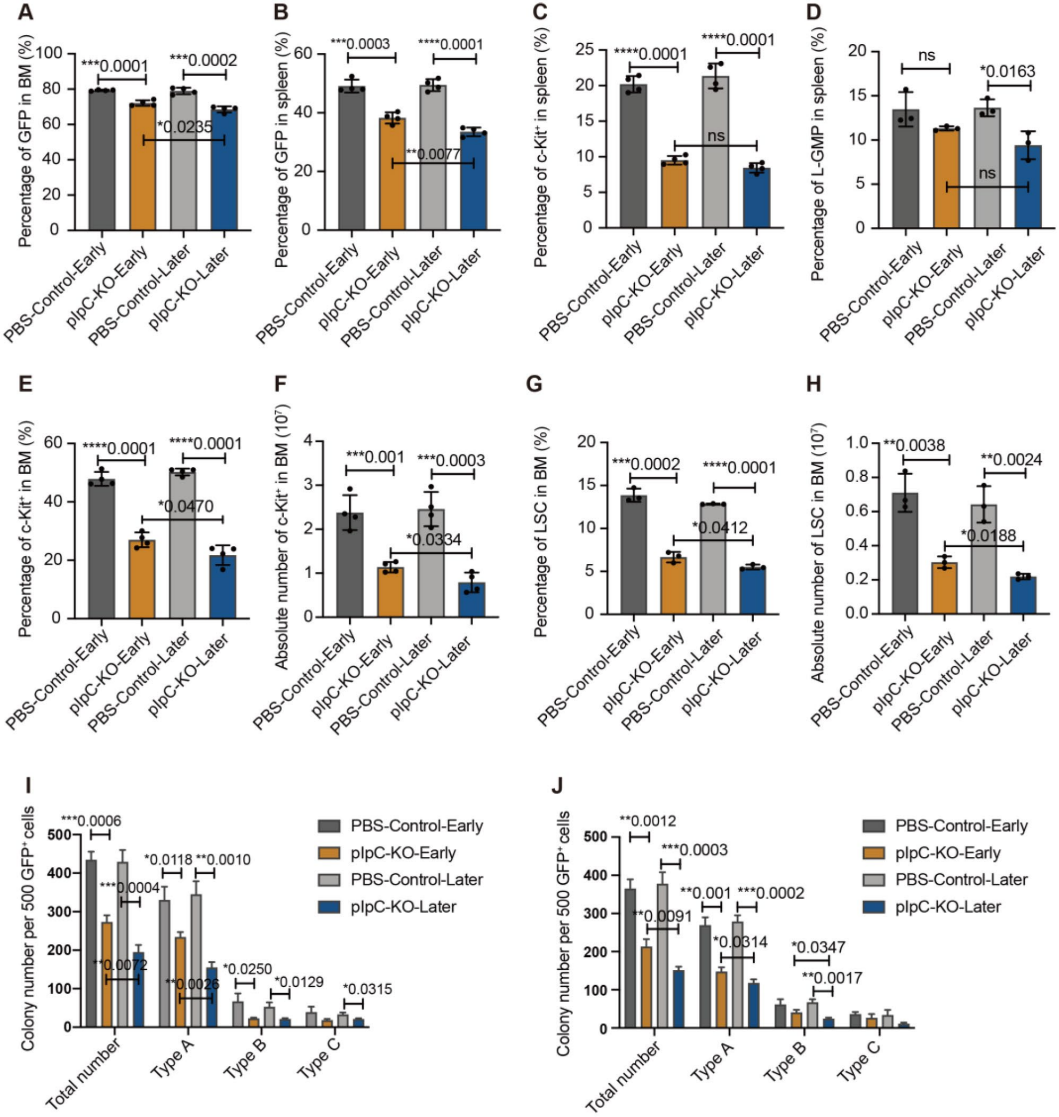
*Phf6* deficiency decreases the activity of leukemia stem cells in AML (**A**-**B**) GFP analysis in BM (**A**) and spleen (**B**). *n* = 4, Student’s *t*-test; (**C**) Analysis of c-Kit^+^ spleen cells. *n* = 4, Student’s *t*-test; (**D**) Analyses of L-GMP in spleen cells. *n* = 3, Student’s *t*-test; (**E**-**F**) The percentage (**E**) and absolute number (**F**) of c-Kit^+^ AML cells in BM. *n* = 4, Student’s *t*-test; (**G**-**H**) The percentage (**G**) and absolute number (**H**) of L-GMP in BM. *n* = 3, Student’s *t*-test; (**I**-**J**) Colony-forming of AML cells from P1 (**I**) and P2 generation (**J**). Type A colonies are very compact without a halo of migrating cells. Type B colonies have a compact center and a halo of single cells. Type C colonies have no center and only single cells. *n* = 3, Student’s *t*-test. *p < 0.05, **p < 0.01, ***p < 0.001, ****p < 0.0001

To investigate the role of *Phf6* in the progression of AML further, we quantified the biological characteristics of AML cells in BM and spleen. The percentage of GFP^+^ leukemia cells decreased in *Phf6* KO groups than in PBS control groups in the BM and spleen (Fig. 5A-B). To determine whether *Phf6* deficiency suppresses AML progression by decreasing LSCs (leukemia stem cells) number and activity, we analyzed the frequency and absolute number of c-Kit^+^ and L-GMP (Lin^−^ c-Kit^+^ IL-7R^−^ Sca-1^−^ CD16/32^+^ CD34^+^) LSCs in BM and spleen. The percentages of c-Kit^+^ and L-GMP LSCs in spleen were significantly decreased in *Phf6* KO groups than PBS control groups (Fig. 5C-D and Supplementary Fig. 4C-D). The percentage and absolute number of c-Kit^+^ AML cells in *Phf6* KO groups were decreased (Fig. 5E-F and Supplementary Fig. 4E), as well as the L-GMP LSCs in BM (Fig. 5G-H and Supplementary Fig. 4F). Moreover, c-Kit^+^ and L-GMP LSCs in the BM of the pIpC-KO-Later group were less than that of the pIpC-KO-Early group (Fig. 5E-H). Further, we investigated the apoptosis and cell cycle of BM AML cells. The percentages of apoptotic c-Kit^+^ AML cells in *Phf6* KO groups were increased (Supplementary Fig. 5A). Flow cytometry analysis of c-Kit^+^ AML cells from pIpC-KO-Later group showed increased G0 phase and decreased G1 phase when compared with the control cell (Supplementary Fig. 5B). We sorted GFP^+^ cells for the serial clone-forming analysis in vitro. We found the total clone numbers in *Phf6* KO groups decreased, as well as the subtypes A, B, and C (Fig. 5I-J). These data collectively suggested that PHF6 loss inhibited the proliferation of LSCs.

### *Phf6* maintains AML progression by inhibiting cell differentiation

**Fig. 6:**
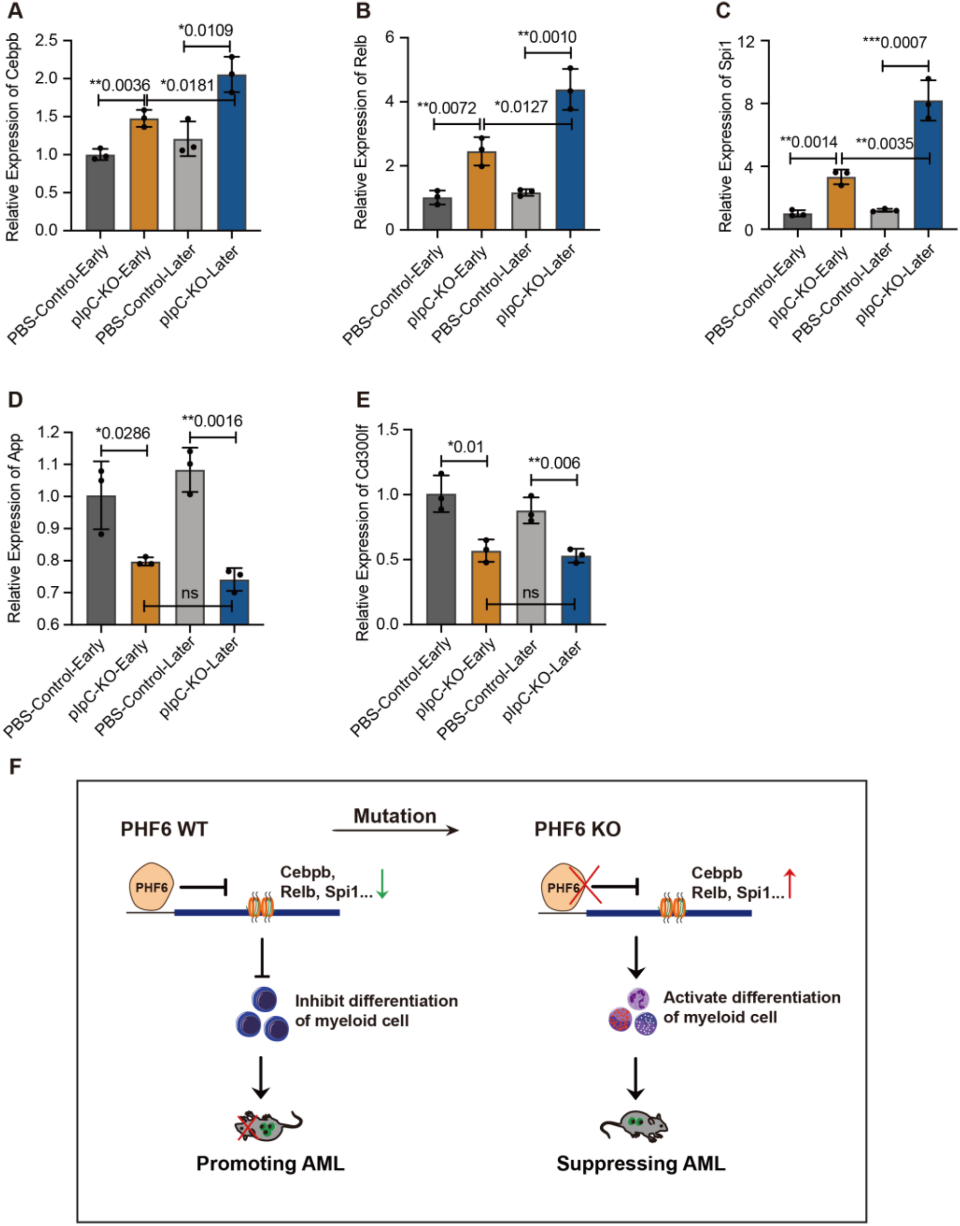
*PHF6* maintains AML progression by inhibiting cell differentiation. (**A**-**C**) Genes related to cell differentiation were examined by RT-PCR. *n* = 3, Student’s *t*-test; (**D**-**E**) Genes related to AML promoting were examined by RT-PCR. *n* = 3, Student’s *t*-test; (**F**) A proposed model for the role of *Phf6* in suppressing AML progression. *p < 0.05, **p < 0.01, ***p < 0.001, ****p < 0.0001

To determine the underlying mechanisms by which PHF6 loss suppresses *MLL::AF9* AML progression, RNA was extracted and inversed to cDNA for RT-PCR. Cebpb, Relb, and Spi1, the predictors of cell differentiation, were increased in *Phf6* KO groups than in PBS control groups (Fig. 6A-C). App and Cd300lf, which were reported to be associated with poor prognosis of AML, were decreased in *Phf6* KO groups compared to the PBS controls (Fig. 6D-E). These results suggested that *Phf6* deficiency suppressed *MLL::AF9-*induced AML development by promoting cell differentiation (Fig. 6F).

## Discussion

AML is the most common malignancy of hematopoietic cells, with poor therapeutic effect and a high recurrence rate [27]. Analyzing the pathogenesis of AML is necessary for developing new therapeutic targets. Large-scale sequencing has found that more than 700 chromosome/gene abnormalities are related to AML [8, 27], but it is still insufficient to analyze the pathogenesis of AML. How co-existing gene mutations regulate the leukemia process and whether the effects of mutations obtained at different stages of the disease vary, these problems have not been resolved and greatly limit treatment strategies. Therefore, it is of great clinical significance to study the role and mechanism of mutant genes in the development of AML. Gene mutations occur gradually in a sequential and multi-step accumulation process [4], ultimately leading to abnormal expansion and stagnation of differentiation in BM cells.

In this study, we found that acquired PHF6 deficiency can inhibit the development of AML, and the inhibition effect at a later stage of the disease seems to be better. According to published studies, PHF6 deletion has no obvious effect on normal hematopoiesis [28], indicating that PHF6 as a drug target is safe. It is reported that in JAK3 mutation or NOTCH1-induced T-ALL, PHF6 deletion can significantly accelerate the development of the disease, indicating that PHF6 plays a tumor-inhibitory role in T-ALL [20, 21]. Interestingly, compared with the high mutation rate (30%) of PHF6 in T-ALL, the mutation rate of PHF6 in AML patients was significantly reduced, with only about 3% mutation rate [17]. In addition, PHF6 mutation in B-ALL was also uncommon. It was reported that PHF6 deficiency significantly inhibited the growth of BCR::ABL1-induced B-ALL mouse leukemia cells and prolonged mice survival time [24]. These results supported different roles of PHF6 in hematopoietic malignancies. Therefore, the function of PHF6 in AML has aroused our interest.

By investigating the clinical database of leukemia patients (data from bloodspot, oncomine and gepia datasets), we found that PHF6 was highly expressed in AML cells and was consistent with *MLL* rearrangement. High expression of PHF6 is significantly related to the poor prognosis of AML patients. By knocking down the PHF6 gene in different AML cells, we found that PHF6 deficiency inhibited the proliferation of these cells. These results suggested that PHF6 may have other functions in different leukemia. In our study, we used the classical *MLL::AF9* fusion gene to construct the AML mouse model and knock out PHF6 gene at different stages of disease development. It was found that PHF6 deletion significantly inhibited the proliferation of leukemic cells and prolonged the survival time of mice. What interests us is that PHF6 knockout at a later disease stage seems to have a better leukemia inhibition effect. We speculate that PHF6 deletion at the later stage may have a more common and effective impact on more leukemia cells.

AML cells display typical characteristics of primitive and immature morphology and have no function of hematopoiesis. It is the goal of researchers to induce AML cells into mature myeloid cells. In the PHF6 KO group, we found that the expression of genes promoting cell differentiation increased (such as Cebpb, Relb, and Spi1) [29–31], while the genes (App, Cd300lf) that support AML progression were decreased [32, 33]. We analyzed cell immunophenotype and morphology and found that the differentiation of AML cells with PHF6 deletion tended to the more mature stage, which meant that such leukemia cells had low malignancy. We also found that the proliferation of c-Kit^+^ leukemia cells with PHF6 deletion was significantly inhibited. These findings suggested a maintenance role of PHF6 in leukemia stem cells (LSCs). We all know LSCs cause AML relapse and drug resistance, so our study shows that inhibiting PHF6 expression may be a potential therapeutic strategy targeting AML patients.

Our study provided a better understanding of PHF6 in regulating AML, but some limitations in this experiment need to be mentioned. Survival curves were statistically significant, but PHF6 KO mice do not have a high survival advantage. To reinforce the PHF6 role in sustaining stemness in AML, treatment with chemotherapy in WT and KO mice models would reveal if PHF6 impairment will sensitize leukemic cells to treatment by inducing differentiation, considering that LSCs often are considered the most chemo-resistant ones. Considering this, we plan to combine AML chemotherapeutic drugs with small molecule drugs targeting the downstream genes of PHF6 to ascertain the therapeutic effect on AML in the later study. What we found interesting but not fully elucidated in our study is why PHF6 deletion at a later stage can better inhibit AML development than in the early stage. We examined some genes (Cebpb, Relb, Spi1) promoting cell differentiation, which showed higher levels when PHF6 was knocked out at the later stage of AML. However, the specific mechanism still needs more research.

## Conclusion

In conclusion, the PHF6 gene plays a pro-oncogenic role in AML in our study and has great disease intervention value. Exploring the impact of acquired PHF6 mutation on the development of AML is consistent with the clinical reality. This research not only enables a better understanding of AML clinical characteristics but also provides a possible scientific basis for PHF6 to become a potential AML therapeutic target.

### Electronic supplementary material

Below is the link to the electronic supplementary material.

Supplementary Material 1: PHF6 loss reduces leukemia stem cell activity in an acute myeloid leukemia mouse model

## Data Availability

The data generated during the current study are available from the corresponding author upon reasonable request.

## Abbreviations

AML: acute myeloid leukemia
BM: bone marrow
FBS: fetal bovine serum
HE: hematoxylin-eosin
HGB: hemoglobin
HSPCs: hematopoietic stem/progenitor cells
i.v.: intravenous injection
Lin^−^: lineage-negative
LSCs: leukemia stem cells
PB: peripheral blood
PHF6: PHD finger protein 6
pIpC: polyriboinosinic acid/polyribocytidylic acid
PLTs: platelets
RBCs: red blood cells
SPF: specific pathogen-free
T-ALL: acute T-lymphocyte leukemia
WBCs: white blood cells
