# Supplementary material for: PHF6 loss reduces leukemia stem cell activity in an acute myeloid leukemia mouse model
Source: Cancer Cell Int. 2024 Feb 9;24:66. doi: 10.1186/s12935-024-03265-w (PMC10858464; doi:10.1186/s12935-024-03265-w)
Supplement: Supplementary file 1 — Supplementary Material 1: PHF6 loss reduces leukemia stem cell activity in an acute myeloid leukemia mouse model [file 12935_2024_3265_MOESM1_ESM.docx]

Supplementary Data for Shengnan Yuan *et al*.: ***PHF6 Loss Reduces Leukemia Stem Cell Activity in an Acute Myeloid Leukemia Mouse Model*** (Including additional methods and 5 additional figures)

**Additional methods**

**Cell lines and culture**

Human AML cell lines used in our experiment included THP-1 and MOLM-13 cells. These two AML cell lines were cultured at 37°C with 5% CO2 in RPMI-1640 medium (Gibco, CA, USA) supplemented with 10% FBS. HEK 293T cell was cultured in a DMEM basic medium with 10% FBS to produce the virus. Experiments were performed within four weeks after fresh viable cells were thawed.

**RNA extraction and quantitative real-time PCR**

RNA was isolated using the TRIZOL-chloroform extraction method. All cDNA samples were synthesized from 500 ng isolated RNA samples using PrimeScript RT Reagent Kit (Perfect Real Time) from Takara. We analyzed the samples in technical triplicates using light cycler®480 and SYBR Green master mix. Samples were measured in technical triplicates. We normalized the samples using the reference gene Gapdh. Sequences of primers are as follows: Cebpb forward 5’- CAAGCTGAGCGACGAGTACA-3’, reverse 5’-GACAGCTGCTCCACCTTCTT-3’.

Relb forward 5’- TGCTCTGTGACAAGGTGCAA-3’, reverse 5’-CATTGACAGTCACGGGCTCT-3’.

Spi1 forward 5’- GGTGGGTGGACAAGGACAAA-3’, reverse 5’-CCGCTGAACTGGTAGGTGAG-3’.

App forward 5’- CCCAAGGCCTCATCATGTGT-3’, reverse 5’-GCTCGTAGATCACACGGAGG-3’.

Cd300lf forward 5’- TCCTCCATGTCCTCCTCTGG-3’, reverse 5’-CCTCTTCAAGGCCTGTCCTG-3’.

**HE staining**

Mice were sacrificed to obtain the femur, spleen, and liver, followed by fixation (decalcification), dehydration, and paraffin embedding. Femurs were sliced into 4 µm thickness and stained with Haematoxylin and Eosin Solution. The pathologic changes in tissues were estimated by an optical microscope.

**Cell cycle analysis**

Cells were firstly stained with flow cytometric antibodies and then fixed with the Cytofix Fixation/Permeabilization Kit (BD Biosciences, New Jersey, USA) according to the manufacturer’s instructions. Cells were stained with Ki67 antibody at room temperature for 30 minutes. Prior to analysis, cells were incubated with 1.50 µM Hoechst 33342 (Invitrogen, Carlsbad, CA) and then analyzed on the LSR Fortessa^TM^ cell analyzer. The antibodies used in the experiment are as follows: PE/Cyanine7 anti-mouse Ki67 (16A8; BioLegend), APC anti-human Ki67 (Ki-67; BioLegend).

**Cell apoptosis analysis**

Cells were firstly stained with flow cytometry antibodies, and then 1 ml Annexin V Binding Buffer was added and washed at 1500 rpm centrifugation for 5 minutes. 5µl Annexin V and 5µl 7-AAD were added and stained at room temperature for 20 minutes. The antibodies used in the experiment are as follows: PE/Cyanine7 anti-Annexin V (BioLegend), PerCP/Cyanine5.5 anti-7-AAD (BD).

**Clone forming assay**

500 GFP^+^ cells were plated in Methocult 3231 (StemCell Technologies, Vancouver, BC, Canada) with mIL-3 (10 ng/ml), mIL-6 (10 ng/ml), GM-CSF (10 ng/ml) and mSCF (50 ng/ml) to assess the clone forming capacity. Cells were incubated at 37 °C for 7 days and manually scored. Each clone forming well represents an independent biological assay, and three biological repeats were plated for each group.

**Western blotting**

Western blot analysis was performed using standard protocols. In brief, proteins resolved on SDS-PAGE were transferred to nitrocellulose membranes. Nitrocellulose blots were incubated at room temperature for 1 hour in blocking buffer (TBST with 5% milk), followed by incubation with indicated antibodies at 4°C overnight. After three 15-minute washes with TBST, the blots were incubated with horseradish peroxidase-conjugated secondary antibody (Cell Signaling Technology, Boston, MA, USA). Immunoreactive bands were visualized using an enhanced chemiluminescence substrate (Bio-Rad, Hercules, CA, USA). Antibodies used in our study were as follows: antibody anti-PHF6 (Abcam, Cambridge, UK) and anti-GAPDH antibody (Cell Signaling Technology, Boston, MA, USA).

**Flow antibodies**

The flow antibodies used in the experiment are as follows: APC anti-mouse TER119 (TER-119; BioLegend), APC anti-mouse Gr-1 (1A8; BioLegend), APC anti-mouse/human CD45R/B220 (RA3-6B2; BioLegend), APC anti-mouse CD3 (17A2; BioLegend), APC anti-mouse CD4 (GK1.5; BioLegend), APC anti-mouse CD8 (53-6.7; BioLegend), APC anti-mouse IL-7R (S18006K; BioLegend), APC anti-mouse Sca-1 (D7 ; invitrogen), PE anti-mouse c-Kit (2B8; BioLegend), BV421 anti-mouse CD34 (SA376A4; BioLegend), APC-Cy7 anti-mouse CD16/32 (93; BioLegend), PE anti-mouse CD3(145-2C11; invitrogen), PE/Cyanine7 anti-mouse Mac-1 (M1/70; BioLegend), PerCP/Cyanine5.5 anti-mouse Gr-1（RB6-8C5; BioLegend).

**Additional figures**

**
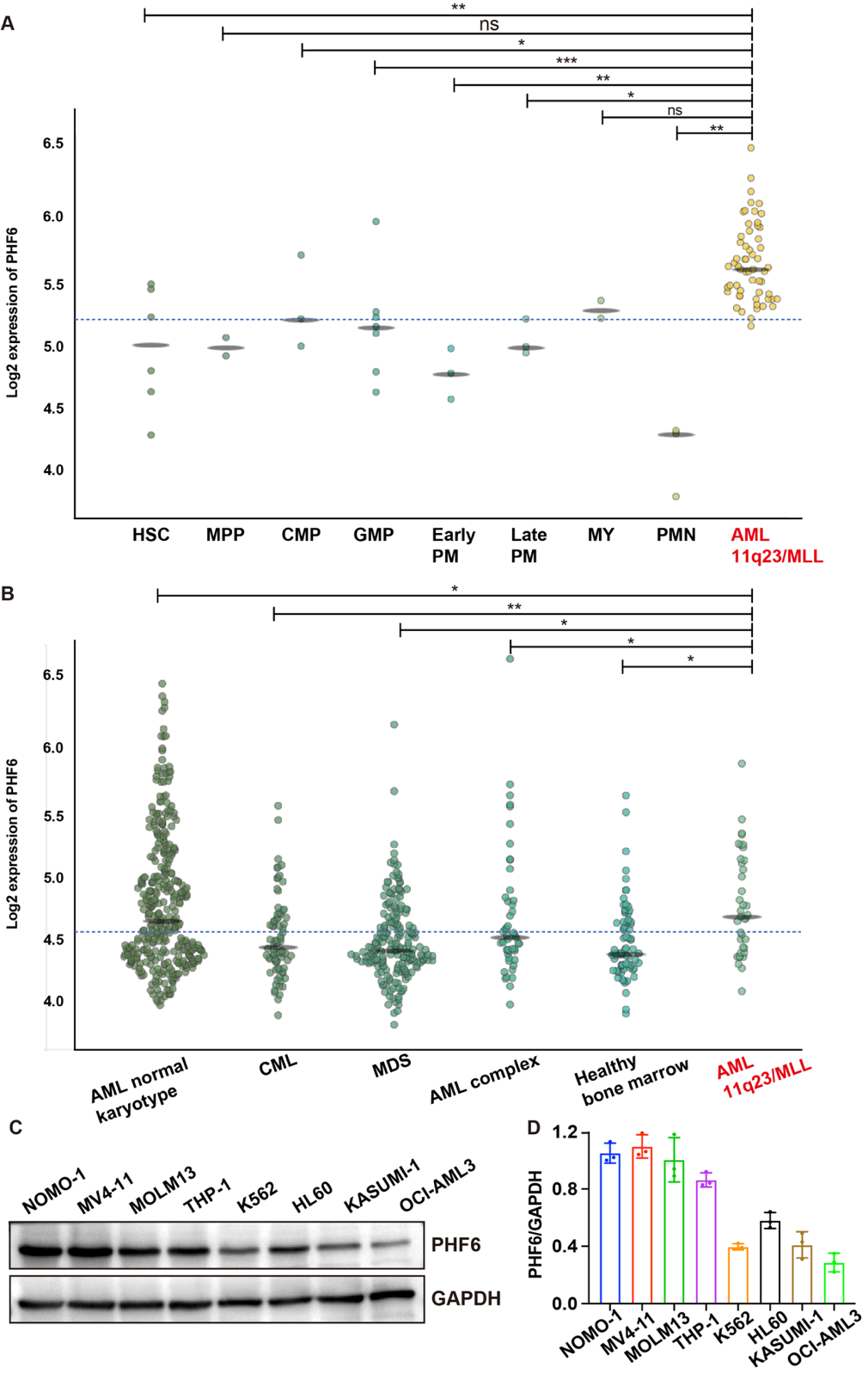
**

**Supplementary Figure 1. High expression of PHF6 in AML with MLL rearrangement** (A) Comparison of PHF6 expression in AML patients with MLL rearrangement (11q23/MLL) and healthy blood cells. Data from bloodspot dataset, www.bloodspot.eu; (B) Comparison of PHF6 expression in AML patients with MLL rearrangement (11q23/MLL), myeloid tumors without MLL rearrangement (including AML normal karyotype, AML complex, CML, and MDS) and in healthy blood cells. Data from bloodspot dataset, www.bloodspot.eu; (C) The expression of PHF6 by western blot in AML cell lines with or without MLL rearrangement; (D) PHF6 expression in AML cells analyzed by densitometry plots. n = 3, Student’s *t*-test. **p* < 0.05, ***p* < 0.01, ****p* < 0.001, *****p* < 0.0001.

**
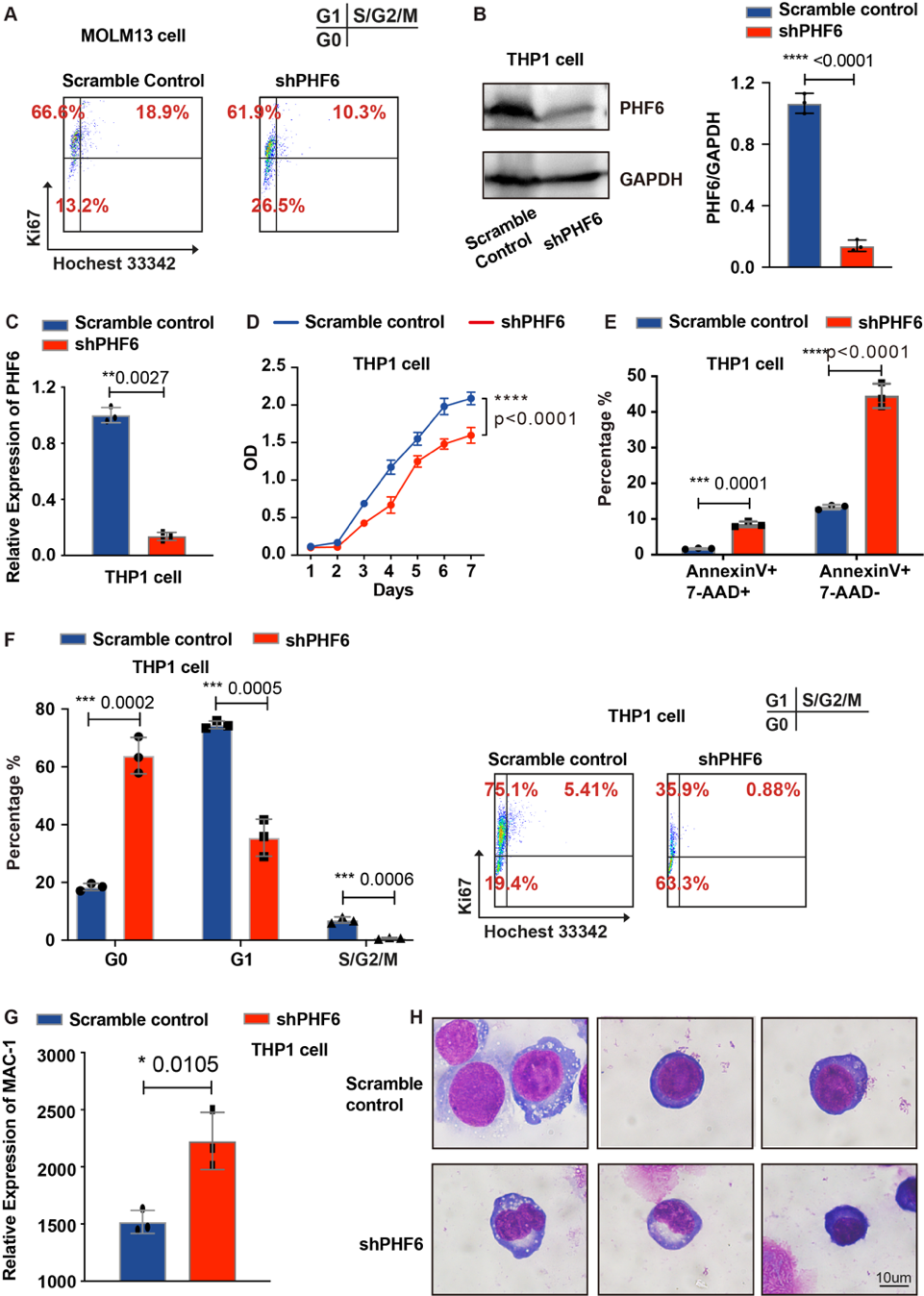
**

**Supplementary Figure 2. Low expression of PHF6 inhibits the proliferation of AML cells** (A) Gating schematic of cell cycle for flow cytometry analysis in MOLM13 cells; (B) PHF6 expression in THP1 cells, as determined by Western Blot (Left) and analyzed by densitometry plots (Right); (C) PHF6 expression in THP1 cells analyzed by QPCR; (D) Examine of cell proliferation by CCK-8. *n* = 8, Student’s *t*-test; (E) Examine of cell apoptosis by annexin V and 7-AAD. *n* = 3, Student’s *t*-test; (F) Analysis of cell cycle in THP1 cells (Left) and gating schematic for the flow cytometry analysis (Right). *n* = 3, Student’s *t*-test; (G) The expression of MAC-1 examined by flow cytometry in THP1 cells; (H) Wright-Giemsa staining of PHF6 knockdown and the control cells (THP1). **p* < 0.05, ***p* < 0.01, ****p* < 0.001, *****p* < 0.0001.


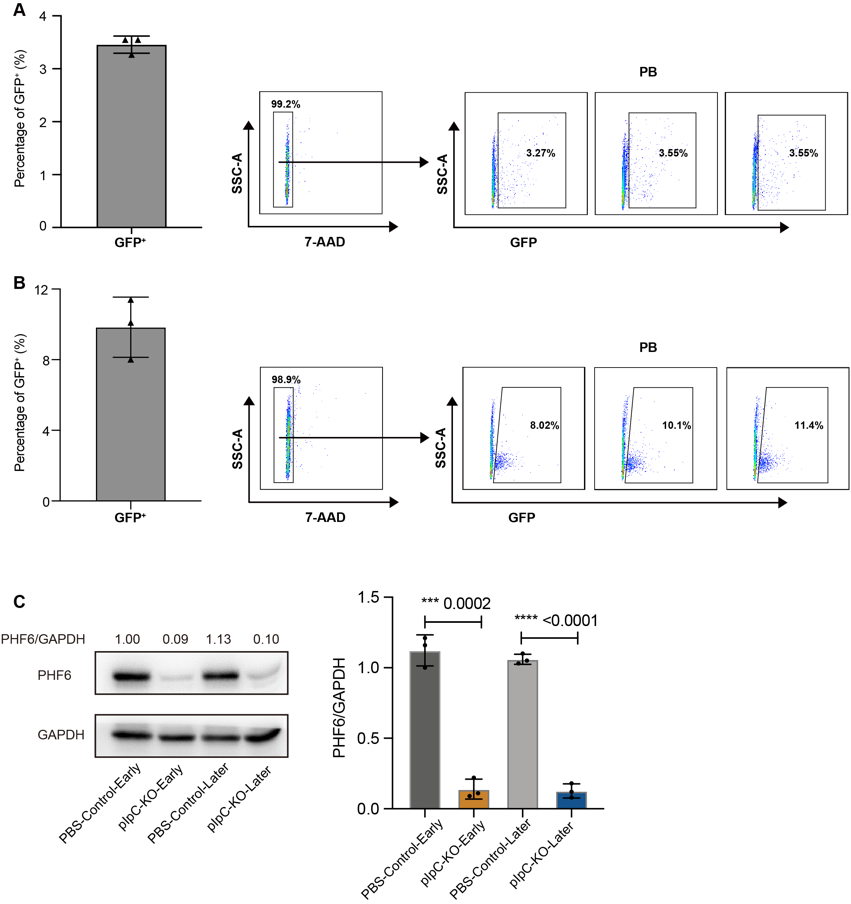


**Supplementary Figure 3. Injection of pIpC and confirm the successful deletion of PHF6** (A-B) The pIpC or PBS was injected when GFP^+^ cells in PB reached 3.5% (the deletion of *Phf6* gene at an early stage) or 10% (the deletion of *Phf6* gene at a later stage) respectively. *n* = 3, Student’s *t*-test; (C) Confirm the deletion of PHF6 by western blot (Left) and analyzed by densitometry plots (Right). **p* < 0.05, ***p* < 0.01, ****p* < 0.001, *****p* < 0.0001.


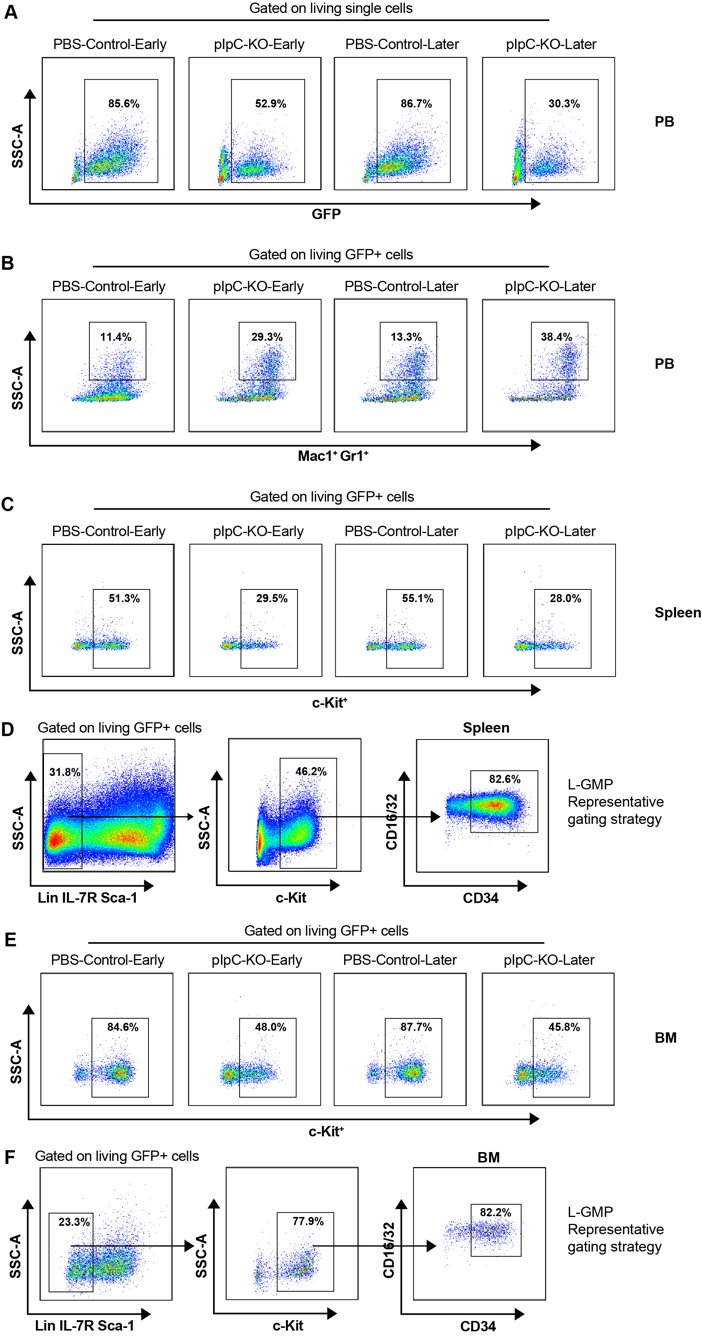


**Supplementary Figure 4. *Phf6* deficiency decreases the activity of leukemia cells in AML** (A-F) Gating schematic for the flow cytometry analysis. (A) Analysis of GFP^+^ cells in the PB. *n* = 4, Student’s *t*-test; (B) Analysis of Mac-1^+^/Gr-1^+^ cells in the PB. *n* = 4, Student’s *t*-test; (C) The percentage of c-Kit^+^ in the spleen. *n* = 4, Student’s *t*-test; (D) Analysis of L-GMP (Lin^−^ c-Kit^+^ IL-7R^−^ Sca-1^−^ CD16/32^+^ CD34^+^) LSCs in the spleen; (E) Analysis of c-Kit^+^ cells in the BM; (F) Analysis of L-GMP LSCs in the BM. *n* = 4, Student’s *t*-test. **p* < 0.05, ***p* < 0.01, ****p* < 0.001, *****p* < 0.0001.


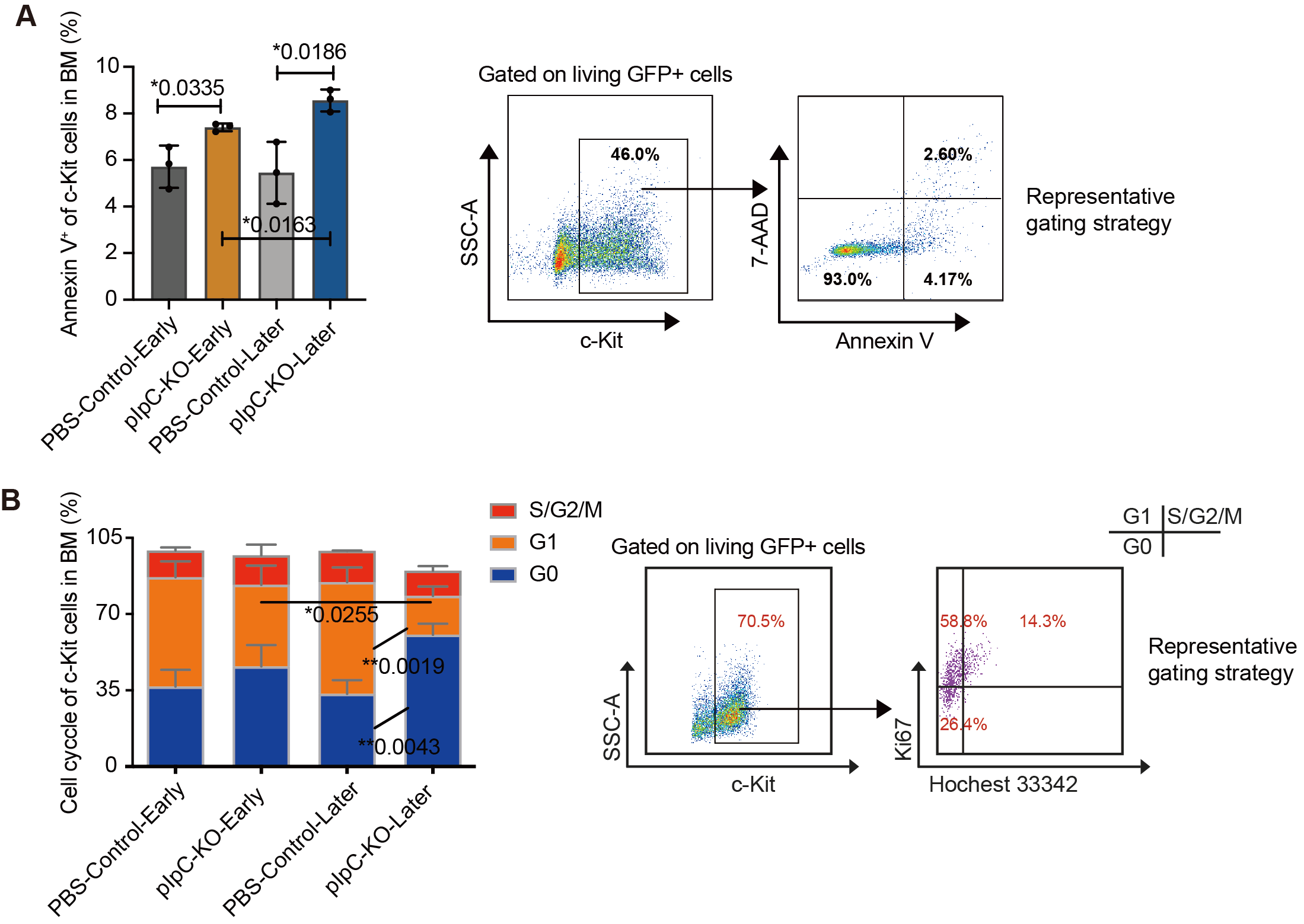


**Supplementary Figure 5. Phf6 deficiency increases the cell apoptosis and decreases the cell cycle of leukemia cells** (A) Analysis of c-Kit^+^ cell apoptosis (Left) and gating schematic for flow cytometry (Right); (B) Analysis of c-Kit^+^ cell cycle (Left) and gating schematic for flow cytometry (Right). n = 3, Student’s t-test. **p* < 0.05, ***p* < 0.01, ****p* < 0.001, *****p* < 0.0001.
